# Gastrointestinal adverse effects of nintedanib and the associated risk factors in patients with idiopathic pulmonary fibrosis

**DOI:** 10.1038/s41598-019-48593-4

**Published:** 2019-08-19

**Authors:** Motoyasu Kato, Shinichi Sasaki, Takahiro Nakamura, Kana Kurokawa, Tomoko Yamada, Yusuke Ochi, Hiroaki Ihara, Fumiyuki Takahashi, Kazuhisa Takahashi

**Affiliations:** 1 Department of Respiratory Medicine, Juntendo University Graduate School of Medicine, Tokyo, Japan; 2 Department of Respiratory Medicine, Juntendo University Urayasu Hospital, Urayasu, Chiba, Japan

**Keywords:** Drug development, Risk factors

## Abstract

Nausea and diarrhea are the most common adverse effects of nintedanib in patients with idiopathic pulmonary fibrosis (IPF). However, the clinical risk factors for these side effects remain unknown. In the present study, we investigated the characteristics of patients who developed gastrointestinal side effects during nintedanib treatment for IPF and determined the risk factors for these side effects. We enrolled 77 patients with IPF who received nintedanib between October 2015 and March 2018. Performance status (PS) as a patient’s general condition, body mass index (BMI), modified Medical Research Council Dyspnea Scale score, severity of IPF at nintedanib initiation, and gastrointestinal toxicity of nintedanib were evaluated. In total, 25 and 27 patients exhibited nausea and diarrhea, respectively, during the follow-up period. A poor PS, low BMI, and full dosage of nintedanib at treatment initiation were risk factors for nausea. A low BMI was a significant risk factor for diarrhea, which could be prevented by combination treatment with nintedanib and prednisolone. In addition, the mean annual rate of decline in forced vital capacity was significantly greater in patients with nausea than in patients without nausea. In conclusion, our findings suggest that patients with a low BMI and/or poor PS and those who receive the full nintedanib dosage at treatment initiation are more susceptible to gastrointestinal adverse effects during nintedanib treatment. Addition of prednisolone to the treatment regimen may prevent the development of diarrhea during treatment.

## Introduction

Nintedanib is often used for the treatment for idiopathic pulmonary fibrosis (IPF)^1^. The INPULSIS trial suggested that treatment with nintedanib at 300 mg/day decreased the annual rate of decline in forced vital capacity (FVC) and acute exacerbations in patients with IPF^2–4^. Nausea, diarrhea, and liver dysfunction are the most common adverse effects of nintedanib, and serious adverse events have been reported in approximately 30% of patients receiving nintedanib treatment. Liver toxicity, including elevated transaminase levels, is also reported to be a significant adverse event. Ikeda *et al*. reported that patients with a body mass index (BMI) ≤22 kg/m^2^ showed a high incidence of liver dysfunction and that a small body surface area was a predictor of liver function disorder during nintedanib treatment^5,6^. In most cases, liver function recovered within 4 weeks. Moreover, most patients who developed mild liver dysfunction had no symptoms. Therefore, increased liver enzyme levels in patients receiving nintedanib treatment may not be clinically significant.

In the INPULSIS trial, 75% of Japanese patients and 62.4% of the total patients developed diarrhea, and 3.5% of Japanese patients discontinued treatment due to severe diarrhea. Furthermore, the incidences of other gastrointestinal symptoms in the Japanese patients such as nausea and appetite loss were 19.7% and 18.4%, respectively^7^. Both diarrhea and nausea continue until nintedanib treatment is discontinued. In patients with IPF, however, discontinuation of nintedanib treatment may worsen the general condition and quality of life. Moreover, the mean FVC was approximately 80% in the INPULSIS cohort; thus, IPF in these patients was probably mild relative to the severity encountered in daily clinical practice. This trial also did not describe the clinical features of patients who developed gastrointestinal symptoms during nintedanib treatment. In the present study, we aimed to investigate the characteristics of patients who developed gastrointestinal side effects during nintedanib treatment for IPF and to determine the risk factors for these side effects.

## Methods

### Study population

We collected data for patients diagnosed with IPF between April 2015 and March 2018 at Juntendo University Hospital and Juntendo University Urayasu Hospital. The patients exhibited definite or possible usual interstitial pneumonia (UIP) patterns on high-resolution computed tomography (HRCT). Patients with secondary interstitial pneumonia, including those with connective tissue disease and/or chronic hypersensitivity pneumonia, were excluded. Definite and possible UIP patterns on HRCT were defined according to the American Thoracic Society (ATS)/European Respiratory Society/Japanese Respiratory Society/Latin American Thoracic Association IPF guideline published in 2011^8^ and HRCT criteria used in the INPULSIS trial^2^. The patients were clinically diagnosed with IPF according to the classification used in the INPULSIS trial. Of patients diagnosed with IPF, those who received nintedanib were enrolled in the present study. The study protocol was approved by the Juntendo University Ethical Committee (number 18-056). The Ethical Committee waived the requirement for informed consent because of the retrospective nature of the study.

### Clinical evaluation

For all enrolled patients, we retrospectively collected and analyzed several clinical parameters, including age, sex, smoking history, performance status (PS), modified Medical Research Council Dyspnea (mMRC) score, BMI, pulmonary function test findings, and GAP index at nintedanib initiation. In addition, the incidence of gastrointestinal adverse effects during nintedanib treatment, including nausea and diarrhea, was recorded for all patients. The general condition of patients was evaluated based on the PS defined by the Eastern Cooperative Oncology Group/World Health Organization (ECOG/WHO), with a PS of >2 representing a poor general condition. mMRC is widely used for the assessment of dyspnea in patients with respiratory diseases, with a score of >2 representing severe dyspnea. BMI was calculated by dividing body weight in kilograms by the square of height in meters. The GAP index was calculated using sex, age, and pulmonary function test findings, including FVC and the diffusing capacity of the lungs for carbon monoxide (DL_CO_). An index of >6 defined severe IPF. We also evaluated FVC every 6 (±3) months during nintedanib treatment, in addition to 6 (±3) months before nintedanib initiation. Changes in FVC were calculated for the following three time periods: 6 months before nintedanib treatment to treatment initiation, treatment initiation to 6 months after, and between 6 and 12 months after treatment initiation. Moreover, gastrointestinal side effects were graded using the Common Terminology Criteria for Adverse Events (CTCAE) ver. 4.0 every 4 weeks following nintedanib initiation.

Because BMI was a significant factor in patients who developed gastrointestinal adverse effects during nintedanib treatment in the INPULSIS trial, we attempted to determine a cut-off value for predicting the development of nausea and diarrhea using receiver operating characteristic (ROC) curve analysis with the highest BMI value. The area under the curve (AUC) was equivalent to the numerator of the Mann-Whitney U statistic comparing marker distributions between patients with nausea and diarrhea and those without these side effects after treatment initiation.

### Statistical analysis

The chi-square test, Fisher’s exact test, and the Wilcoxon two-sample test were used to evaluate the frequencies of gastrointestinal adverse effects and to compare patient characteristics between the adverse effects and no adverse effects groups. Parametric and non-parametric data were compared using Student’s t-test and the Mann-Whitney U test, respectively. Differences in median survival times (MST) were analyzed using the log-rank test. The sensitivity, specificity, and diagnostic accuracy of the cut-off BMI value were determined. Cox proportional hazard analysis was used to calculate hazard ratios (HRs), and univariate and multivariate logistic regression analyses were used to determine the risk factors for the gastrointestinal adverse effects. A p-value of <0.05 was considered statistically significant. All statistical analyses were performed using SPSS version 19.0 for Windows (Chicago, IL, USA).

## Results

### Patient characteristics

Between April 2015 and March 2018, 89 Japanese patients received nintedanib treatment at these hospitals. Of these 89, 77 patients were clinically diagnosed with IPF based on the UIP pattern on HRCT. The characteristics of these patients are shown in Table 1. The median age was 71 (range: 46–88) years. There were 12 (15.6%) women, 11 (14.3%) non-smokers, and 24 (31.2%) patients with a poor PS (2–4). The mean BMI was 22.9 ± 0.72 kg/m^2^. Twenty-two (73.3%) patients lost body weight at the initiation of nintedanib. The mean FVC, %FVC, and %DLco were 2.19 ml, 65.42%, and 29.61%, respectively (Table 1). For 26 patients, nintedanib treatment was started at a dosage of 200 mg/day by advanced age and/or poor PS.

**Table 1:** Characteristics of patients with idiopathic pulmonary fibrosis treated with nintedanib.

Characteristics	Present study	Nintedanib group in INPULSIS (Overall)	Nintedanib group in INPULSIS (Japanese)
	n = 77	n = 638	n = 76
**Age**	71.6 ± 8.3	66.6 ± 8.1	68.4 ± 7.6
**Sex**			
Female/Male	12/65	131/507	14/62
BMI	22.7 ± 3.95	28.1 ± 4.6	24.4 ± 3.4
**Smoking history**			
No/Yes	11/66	174/464	10/66
**PS**			
0–1/2–4	53/24	—	—
**mMRC**			
0–1/2–4	36/41	—	—
**HRCT pattern**			
Possible UIP/Definite UIP	24/53	213/425	—
**GAP index**			
1–5/6–9	41/36	—	—
**Prior treatment (Steroid)**			
No/Yes	52/25	402/136	67/9
**Initiation dose of nintedanib**			
300 mg/200 mg	51/26	638/0	76/0
FVC (mL)	2.19 ± 0.72	2.71 ± 0.76	2.42 ± 0.67
%FVC (%)	65.42 ± 19.24	79.7 ± 17.6	80.9 ± 16.6
%DLco (%)	29.61 ± 12.04	47.4 ± 13.5	44.6 ± 11.4

BMI: body mass index, PS: performance status, mMRC: modified Medical Research Council Dyspnea Scale, HRCT: high-resolution computed tomography, UIP: usual interstitial pneumonia, FVC: focal vital capacity, DLco: diffusing capacity of the lungs for carbon monoxide.

Twenty-five patients received prednisolone before the initiation of nintedanib treatment. The difference in patient baseline characteristics between patients who received nintedanib only and those who received both nintedanib and prednisolone is shown in Table S1. There were no significant differences in patient backgrounds, including age, sex, smoking history, PS, GAP index, BMI, HRCT pattern, pulmonary function test results (FVC and DLco), and nintedanib initiation dosage (300 or 200 mg) between the two groups in our cohort. In concomitant medication, although the percentage of patients who received proton pump inhibiters was significantly higher among those who received both prednisolone and nintedanib than among those who received nintedanib only (61.5% vs 28%, p = 0.007), there was no significant difference in the percentage of patients who received intestinal drugs between the two groups (70.6% vs 64%, p = 0.910). Among these patients, 18 received prednisolone due to the progression of interstitial pneumonia before initiation of nintedanib treatment. In most of these patients, treatment with nintedanib was started over a year prior. Among the 18 patients who had received prednisolone, eight of them received it because the clinician considered that their pathogenesis of interstitial pneumonia associated with auto-immune features. Although these patients had possible serum auto-immune antibodies or physical findings related to those of autoimmune diseases, the clinicians could not diagnose these patients as all autoimmune diseases. The other eight of these 18 patients had received small amounts of corticosteroids a long time prior to the publishing of the international IPF treatment statement. Among remaining seven patients, corticosteroid treatment was initiated in five for drug-induced lung toxicity superimposed on IPF. The other two patients received prednisolone for diseases other than lung disease. We started treating with nintedanib when the prednisolone dose was within 20 mg/day. In the INPULSIS trial, we were able to combine nintedanib with prednisolone (15 mg/day or less)^4^. Thus, there was not much difference between our data and the INPULSIS trial in the allowed prednisolone dosage and the effect of prednisolone on nintedanib treatment.

### Gastrointestinal adverse effects

The gastrointestinal adverse effects of nintedanib are summarized in Table 2. Twenty-five patients (32.5%) developed grade 2 or worse nausea. Although all 25 patients received prokinetic agents, 13 (16.8%) required treatment discontinuation. Twenty-seven patients (35.1%) developed grade 2 or worse diarrhea, and although all these patients received anti-flatulence or anti-diarrhea drugs, 10 (12.9%) required treatment discontinuation after diarrhea onset.

**Table 2:** Gastrointestinal side effects of nintedanib in patients with idiopathic pulmonary fibrosis.

Adverse events	Number	Incidence
Nausea	25	32.5%
Nausea (Discontinuation)	13	16.8%
Diarrhea	27	35.1%
Diarrhea (Discontinuation)	10	12.9%

### Cut-off BMI value

**Figure 1:**
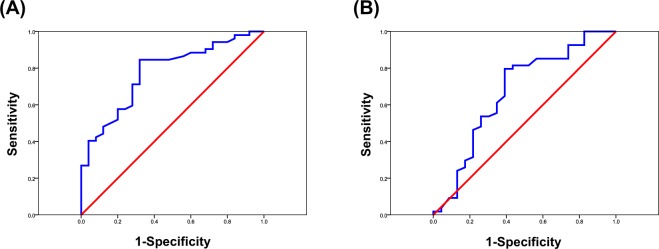
Receiver operating characteristic (ROC) curve analysis to determine cut-off body mass index (BMI) values for predicting the development of nausea and diarrhea in patients with idiopathic pulmonary fibrosis receiving nintedanib treatment. Sensitivity is plotted on the y-axis and specificity on the x-axis. (**A**) The area under the curve (AUC) is equivalent to the numerator of the Mann-Whitney U statistic comparing the marker distributions for the development of nausea [AUC, 0.873; 95% confidence interval (CI), 0.784–0.962; p = 0.001]. The optimal cut-off value is 21.6 kg/m^2^, with sensitivity, specificity, and likelihood ratios of 68.0%, 84.7%, and 4.533, respectively. (**B**) AUC is equivalent to the numerator of the Mann-Whitney U statistic comparing the marker distributions for the development of diarrhea (AUC, 0.668; 95% CI, 0.502–0.834; p = 0.036). The optimal cut-off value is 21.6 kg/m^2^, with sensitivity, specificity, and likelihood ratios of 60.8%, 80.1%, and 3.156, respectively.

Regarding the cut-off BMI value, the AUC was 0.873 [95% confidence interval (CI), 0.784–0.962; p = 0.001] for nausea development and 0.668 (95% CI, 0.502–0.834; p = 0.036) for diarrhea development. The optimal cut-off value was 21.6 for both nausea and diarrhea (Fig. 1).

### Association between pulmonary function and gastrointestinal adverse effects

**Figure 2:**
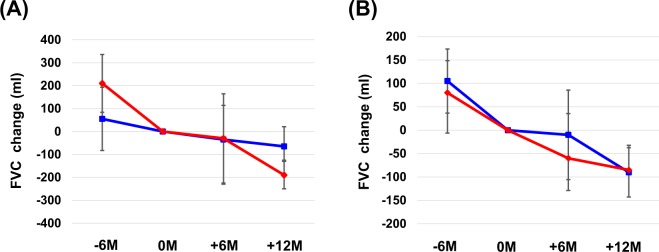
Changes in forced vital capacity (FVC) during nintedanib treatment for idiopathic pulmonary fibrosis. Differences in the annual rate of FVC decline between patients with nausea and those without (**A**) and between patients with diarrhea and those without (**B**). The red line shows the average FVC decline for patients with the adverse effect, while the blue line shows the average FVC decline for patients without the adverse effect.

Nausea development was significantly associated with the baseline DLco, while diarrhea development was significantly associated with the baseline FVC. (Table S2) The average annual rate of decline in FVC was significantly greater in patients with nausea (190 ml/year) than in those without nausea (65 ml/year; p = 0.012; Fig. 2A). In contrast, no significant difference was observed between patients with diarrhea and those without diarrhea (85 ml/year vs. 90 ml/year, respectively; p = 0.683; Fig. 2B).

### Comparisons between the adverse effects and no adverse effects groups, the risk factors for gastrointestinal adverse effects, and prognosis

Monovariate analysis showed that the nausea incidence was significantly higher for patients with a poor PS, high mMRC, high GAP index, and/or low BMI than for patients with a satisfactory PS, low mMRC, low GAP index, and/or high BMI (Table 3). The incidence of diarrhea was significantly higher for patients with a high GAP index and/or low BMI than for patients with a low GAP index and/or high BMI (Table 4). However, the incidence of diarrhea was significantly lower for patients who received both prednisolone and nintedanib than for those who received nintedanib only.

**Table 3:** Comparison between patients with nausea and those without nausea during nintedanib treatment for idiopathic pulmonary fibrosis and risk factors for nausea.

Characteristics	Overall	No	Yes	Monovariate analysis			Multivariate analysis		
				OR	95% CI	*p*	OR	95% CI	*p*
	77	52	25						
**Age**				0.508	0.187–1.383	*0.224*			
≤74	44	27	17						
≥75	33	25	8						
**Sex**				1.534	0.376–6.249	*0.741*			
Female	12	9	3						
Male	65	43	22						
**Smoking history**				1.333	0.321–5.528	*0.988*			
No	11	8	3						
Yes	66	44	22						
**PS**				3.876	1.364–11.019	*0.015*	10.048	1.711–59.001	*0.011*
0–1	55	42	13						
2–4	22	10	12						
**mMRC**				2.769	1.014–7.564	*0.044*	1.876	0.272–7.219	*0.255*
0–1	52	39	13						
2–4	25	13	12						
**GAP index**				3.375	1.250–9.115	*0.014*	3.058	0.736–12.705	*0.124*
≤5	46	36	10						
≥6	31	16	15						
**BMI**				15.011	3.727–60.372	<*0.001*	10.841	2.644–44.448	*0.001*
<21.6	39	35	4						
≥21.6	19	7	12						
**PSL combination**				0.549	0.186–1.611	*0.312*	0.861	0.184–4.028	*0.849*
No	52	33	19						
Yes	25	19	6						
**Nintedanib dose**				3.400	0.864–5.790	*0.065*	22.965	2.754–191.464	*0.004*
300 mg/day	51	31	20						
200 mg/day	26	21	5						

OR: odds ratio, CI: confidence interval, PS: performance status, mMRC: modified Medical Research Council Dyspnea Scale, BMI: body mass index, PSL: prednisolone.

**Table 4:** Comparison between patients with diarrhea and those without diarrhea during nintedanib treatment for idiopathic pulmonary fibrosis and risk factors for diarrhea.

Characteristics	Overall	No	Yes	Monovariate analysis			Multivariate analysis		
				OR	95% CI	*p*	OR	95% CI	*p*
	77	50	27						
**Age**				1.540	0.205–1.434	*0.215*			
≤74	44	26	18						
≥75	33	24	9						
**Sex**				1.393	0.137–1.658	*0.238*			
Female	12	6	6						
Male	65	44	21						
**Smoking history**				0.389	0.107–1.420	*0.144*			
No	11	5	6						
Yes	66	45	21						
**PS**				2.438	0.880–6.749	*0.082*	1.564	0.381–6.507	*0.531*
0–1	56	39	16						
2–4	21	11	11						
**mMRC**				1.768	0.660–4.768	*0.255*	1.171	0.260–5.265	*0.837*
0–1	52	36	16						
2–4	25	14	11						
**GAP Index**				6.241	1.277–9.019	*0.012*	1.829	0.546–6.127	*0.328*
≤5	46	35	11						
≥6	31	15	16						
**BMI**				4.432	1.611–12.191	*0.003*	3.460	1.044–11.467	*0.042*
<21.6	52	39	12						
≥21.6	25	11	15						
**PSL combination**				0.240	0.072–0.798	*0.015*	0.241	0.059–0.995	*0.049*
No	52	29	23						
Yes	25	21	4						
**Nintedanib dose**				1.286	0.468–3.532	*0.626*	1.170	0.321–4.265	*0.812*
300 mg/day	54	36	18						
200 mg/day	23	14	9						

OR: odds ratio, CI: confidence interval, PS: performance status, mMRC: modified Medical Research Council Dyspnea Scale, BMI: body mass index, PSL: prednisolone.

Multivariate analysis for the estimation of risk factors for nausea and diarrhea was performed with the following variables: PS, mMRC score, GAP index, BMI, prednisolone treatment, and initiation dose of nintedanib. A low PS, low BMI, and a nintedanib initiation dosage of 300 mg/day were significant risk factors for nausea (Table 3), while a low BMI was a significant risk factor for diarrhea (Table 4). Combination treatment with both prednisolone and nintedanib significantly decreased the risk of diarrhea.

**Figure 3:**
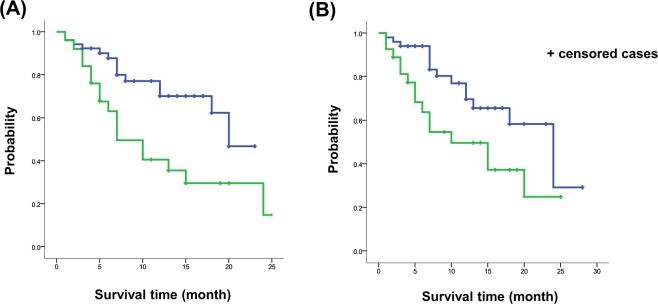
Survival times during nintedanib treatment for idiopathic pulmonary fibrosis. Differences in median survival times between patients with nausea and those without (**A**) and between patients with diarrhea and those without (**B**). The green line represents the survival curve for patients with the adverse effect, while the blue line represents the curve for patients without the adverse effect.

Eighteen (23.4%) patients who received nintedanib died during the follow-up period. Differences in MST between the adverse effects and no adverse effects groups are shown in Fig. 3. MST was significantly shorter for patients with nausea (7 months; 95% CI, 3.390–10.610) or diarrhea (10 months; 95% CI, 3.003–16.997) than for those without nausea (20 months; 95% CI, 11.306–28.964; HR, 7.397; p = 0.007; Fig. 3A) or diarrhea (24 months; 95% CI, 15.433–32.567; HR, 5.240; p = 0.022; Fig. 3B).

## Discussion

To our knowledge, the present study is the first to evaluate the risk factors for gastrointestinal toxicity during nintedanib treatment for IPF. The six main findings were as follows. First, the optimal cut-off BMI value for predicting nausea and diarrhea during treatment was 21.6 kg/m^2^. Second, a poor PS, a low BMI, and nintedanib treatment initiation at the full dosage (300 mg/day) were risk factors for nausea during treatment. Third, combination treatment with nintedanib and prednisolone significantly prevented diarrhea. Fourth, a low BMI was a significant risk factor for the development of diarrhea. Fifth, the annual rate of FVC decline was significantly influenced by nausea. Sixth, a low initial dosage (200 mg/day) was a significant protective factor against the development of nausea.

Azuma *et al*. explained that Japanese patients are more susceptible to hepatic disorders, as they have lower BMI than Caucasians^7^. Therefore, an initial nintedanib dosage of 300 mg/day may be too high for Japanese patients as per another study, which recommended a dosage of 200 mg/day for this population^9^. In the present study, an initial dosage of 200 mg/day significantly decreased the incidence of nausea in patients with IPF. Accordingly, we recommend this initial dosage for patients with low BMI and poor PS measures.

The PS is generally used to evaluate the general condition of patients with neoplasms. However, this parameter is rarely used to evaluate the condition of patients with interstitial pneumonia. Nintedanib is a small-molecule tyrosine kinase and inhibits platelet-derived growth factor (PDGFR), vascular endothelial growth factor (VEGFR), and fibroblast growth factor receptors (FGFR)^10–12^ and was developed as an anti-cancer agent for patients with non-small cell lung cancer^13,14^. We therefore evaluated PS in our patients before nintedanib initiation. For patients with interstitial pneumonia, mMRC is generally used for the evaluation of general condition. We analyzed the mMRC score as well as PS before nintedanib treatment initiation and found that PS, but not the mMRC score, was a significant risk factor for gastrointestinal toxicity. Thus, PS may be a more appropriate parameter for evaluation of the toxicity of anti-fibrotic agents.

Next, we analyzed baseline body weight and BMI at the initiation of nintedanib treatment; however, we did not evaluate the change in body weight or BMI. In the INPULSIS trial, the incidence of weight loss was reported to be 6–8% as an adverse effect of nintedanib. However, we usually observe weight loss in patients with progressive IPF; thus, it was difficult to differentiate between weight loss due to the adverse effects of nintedanib and that due to disease progression. In this study, we focused on the gastrointestinal adverse effects induced by nintedanib. We therefore excluded weight loss from the evaluation in our study.

Prednisolone is used for the treatment of patients with interstitial pneumonia. It was reported that combination therapy with prednisolone, azathioprine, and N-acetylcysteine increased the mortality and hospitalization rates for patients with IPF because of an increase in the incidence of infections^15^. Since the publication of these findings, steroids are generally not used together for IPF treatment. However, 25 patients in our study received steroid therapy. We found that the incidence of diarrhea was significantly lower in these patients than in those who did not receive prednisolone. There were no significant differences in patient characteristics, except for the concomitant treatment with proton pump inhibitor; therefore, combination treatment with a steroid and nintedanib may prevent nintedanib-associated diarrhea.

The mechanism of the development of nintedanib-induced diarrhea is still unknown. There have been many reports on the mechanisms of many kinds of drug-induced intestinal damage^16,17^; here, we propose two potential mechanisms for the development of diarrhea induced by nintedanib. One potential mechanism is based on receptor inhabitation; the other is the direct inflammation of the intestinal epithelium by nintedanib or decomposition products.

Nintedanib is a triple receptor (PDGFR, VEGFR, and FGFR) tyrosine kinase inhibitor. We should have investigated which receptors were associated with the development of diarrhea in this study. It would have been difficult, however, to evaluate how each individual receptor tyrosine kinase inhibitor leads to the development of diarrhea, as there were no PDGFR- or FGFR-only targeted marketing medicine now.

The VEGFR tyrosine kinase inhibitor was known as ramucirumab for non-small cell lung, gastric, and ovarian cancer. Ramucirumab monotherapy was used for progressive gastric cancer only. The incidence of ramucirumab-induced diarrhea was reported to be approximately 14% in gastric cancer patients^18^. VEGFR inhibition was reported to be associated with ischemia and hypoxia in the bowel mucosa and to follow a pathogenesis similar to that of ischemic colitis by inhibition of c-KIT as well as imatinib^19,20^. Epidermal growth factor receptor (EGFR) tyrosine kinase inhibitor (EGFR-TKI) is used for non-small cell lung cancer, and one of the major adverse effects in EGFR-TKI is diarrhea. EGFR is expressed in intestinal epithelium; therefore, inhibition of EGFR was reported to lead to the development of diarrhea. Recently, EGFR upregulation in IPF fibroblasts has been shown to be blocked by nintedanib. The FGF pathway is reported to interplay with EGF signaling; thus, nintedanib causes the development of diarrhea through the inhibition of FGFR^21^. However, it has never been reported that steroid treatment improves or prevents the development of diarrhea by inhibition of these pathways or mechanisms.

On the other hand, direct mucosal damage is one of the mechanisms of the development of diarrhea^16^. Nintedanib or decomposition products may directly injure intestinal epithelium and lead to the development of diarrhea. If this inflammation is similar to inflammatory bowel disease, corticosteroid treatment may improve intestinal inflammation and related symptoms, including stomach ache and diarrhea. However, the above hypotheses were not proven by human data or *in vivo* experiments; thus, it would be necessary to use an *in vivo* model to clarify the mechanism of the development of diarrhea and the reason why corticosteroid treatment prevents or improves diarrhea induced by nintedanib. We also should re-evaluate the clinical effect of prednisolone for the improvement or prevention of nintedanib-induced diarrhea in IPF patients by prospective study.

The FVC decline was significantly greater in patients who developed nausea than in those who did not experience these side effects. Almost patients who developed nausea could not consume food or medications. Therefore, we decided to interrupt treatment and resume it after the resolution of nausea, and we lowered the dosage to 200 mg/day. In contrast, most patients with diarrhea could continue to consume medications orally. Thus, a decrease in nintedanib dosage due to the side effect of nausea may have attenuated the effect of the drug against a decline in FVC. Moreover, baseline pulmonary function and baseline BMI were lower in our cohort than in the INPULSIS cohort (Table 1) because our study was retrospective and conducted in a real-world setting. Specifically, although the FVC baseline was 50% or more of the predicted value in patients in the INPULSIS trial, one fourth of patients had a FVC within 50% in our study, a poor PS, and a low BMI. In other words, worsening general condition may be associated with progression of IPF. Thus, there was a difference in baseline background characteristics between patients in the INPULSIS cohort and our patients. Abe, *et al*. reported on evaluating the effect of nintedanib for the severe grade of IPF^22^. In this publication, the patients with a severe grade of IPF could not continue to receive nintedanib, and survival times were significantly shorter in patients who could not continue nintedanib treatment than in patients who could continue treatment. In this study, although there was no data on the change in FVC after discontinuation of nintedanib, we consider that the shorter survival times in this study were similar to the decline in FVC for the negative influence of the discontinuation of nintedanib treatment. The incidence of serious adverse events is significantly higher in patients with a FVC ≤50% predicted than in those with a FVC >50% predicted (63.4% vs. 39.3%) in the INPULSIS-on trial^23,24^. The incidence of adverse events leading to treatment discontinuation was higher in patients with a FVC <50% compared to that in patients with a FVC >50% (22.5% vs 41.5%, respectively). Therefore, low baseline FVC may be associated with the development of adverse effects and the discontinuation of nintedanib treatment in these published data. In our study, the patients with a poor PS or low BMI easily developed nausea compared to those with a good PS or high BMI. Severe grade (GAP index >6) was not a significant risk factor for the development of nausea by multivariate analysis in our study; the incidence of nausea was significantly higher in patients with high grade severity than in patients with low grade severity. (Table 3) A poor PS and low BMI, in other words, a worsening general condition, may be associated with progression of IPF^22^. Moreover, the development of nausea is associated with a poor PS and low BMI, and the decline in FVC in patients with nausea was significantly larger than that in patients without nausea in this study. The many patients with progressive IPF cannot continue to receive nintedanib for a long time based on Abe’s publication. Therefore, we consider that the decline in FVC in patients with poor general condition, including poor PS and low BMI, was larger than that in patients with a good PS or high BMI in our study. These results were different from those in the INPULSIS trial. This difference in the decline in FVC between two cohorts may reflect the difference between clinical trials and real-world data. Real-world data include more patients with poorer general condition and low pulmonary function; thus, we may start to treat IPF patients with nintedanib beginning at the early stage of IPF.

This study has several limitations. The first was its small sample size and retrospective design. Although nintedanib became commercially available 3 years ago, the number of patients who received the drug in our institutes was limited. Further prospective studies with larger sample sizes are needed to clarify our findings. Nevertheless, our study provides important information about the gastrointestinal toxicity of nintedanib in the clinical setting. Second, compared with the INPULSIS cohort, the backgrounds of our patients were heterogeneous. The patients’ background data, including BMI and pulmonary function, were lower in our cohort than in the INPULSIS trial. In other words, some of our results differ from those of the INPULSIS trial. Furthermore, IPF may have been more severe in our study population, which included more elderly patients, worse PS measures, and lower BMI compared to the INPULSIS cohort. These differences are considered to come from differences between real-world data and clinical trials. Therefore, the prediction error in our study could be larger than that in the INPULSIS trial.

## Conclusion

Our findings suggest that a low BMI is a risk factor for nausea and diarrhea during nintedanib treatment for IPF. In addition, a poor PS and a full initial dosage of nintedanib (300 mg/day) can result in nausea during treatment. The addition of a steroid to the treatment regimen may prevent diarrhea. Clinicians should plan nintedanib treatment after careful consideration of these risk factors.

## Supplementary information

Supplementary Information
