# Author Correction: Gastrointestinal adverse effects of nintedanib and the associated risk factors in patients with idiopathic pulmonary fibrosis

**DOI:** 10.1038/s41598-020-69088-7

**Published:** 2020-07-16

**Authors:** Motoyasu Kato, Shinichi Sasaki, Takahiro Nakamura, Kana Kurokawa, Tomoko Yamada, Yusuke Ochi, Hiroaki Ihara, Fumiyuki Takahashi, Kazuhisa Takahashi

**Affiliations:** 1 Department of Respiratory Medicine, Juntendo University Graduate School of Medicine, Tokyo, Japan; 2 Department of Respiratory Medicine, Juntendo University Urayasu Hospital, Urayasu, Chiba Japan

Correction to: *Scientific Reports* https://doi.org/10.1038/s41598-019-48593-4, published online 19 August 2019

This Article contained errors in Tables 1, 3 and 4 due to incorrectly written code.

In Table 1, the totals reported for PS 0-1/2-4, mMRC 0-1/2-4 and GAP index 1-5/6-9 under the subheading “Present study n = 77”, are incorrect.

The correct totals should read 55/22, 52/25 and 46/31 respectively.

As a result, in the Results section under the subheading ‘Patients characteristics’,

“The median age was 71 (range: 46–88) years. There were 12 (15.6%) women, 11 (14.3%) non-smokers, and 24 (31.2%) patients with a poor PS (2–4). The mean BMI was 22.9 ± 0.72 kg/m^2^.”

should read:

“The median age was 71 (range: 46–88) years. There were 12 (15.6%) women, 11 (14.3%) non-smokers, and 22 (28.6%) patients with a poor PS (2–4). The mean BMI was 22.7 ± 3.95 kg/m^2^.”

In Table 3, the totals reported for BMI < 21.6 and BMI ≥ 21.6 are incorrect.

The correct totals should read 25, 12, 13 and 52, 40, 12 respectively.

In Table 4, the totals reported for PS 0-1/2-4, BMI < 21.6, BMI ≥ 21.6 and Nintedanib dose for 300 mg/day and 200 mg/day are incorrect.

The correct totals should read 55, 39, 16 and 22, 11, 11 and 25, 10, 15 and 52, 40, 12 and 51, 34, 17 and 26, 16, 10 respectively.

