# Supplementary material for: Gastrointestinal adverse effects of nintedanib and the associated risk factors in patients with idiopathic pulmonary fibrosis
Source: Sci Rep. 2019 Aug 19;9:12062. doi: 10.1038/s41598-019-48593-4 (PMC6700168; doi:10.1038/s41598-019-48593-4)
Supplement: Supplementary file 1 — Supplementary Information [file 41598_2019_48593_MOESM1_ESM.docx]

**Supplementary information**

**Gastrointestinal adverse effects of nintedanib and the associated risk factors in patients with idiopathic pulmonary fibrosis**

**Authors’ full names:** Motoyasu Kato^1^, Shinichi Sasaki^1,2^, Takahiro Nakamura^1^, Kana Kurokawa^1^, Tomoko Yamada^1^, Yusuke Ochi^1^, Hiroaki Ihara^1^, Fumiyuki Takahashi^1^, Kazuhisa Takahashi^1^

**Authors’ affiliations:** ^1^ Department of Respiratory Medicine, Juntendo University Graduate School of Medicine, ^2^ Department of Respiratory Medicine, Juntendo University Urayasu Hospital

**Supplementary Methods**

**Assessment of nausea and diarrhoea during nintedanib treatment for idiopathic pulmonary fibrosis**

Nausea and diarrhoea were graded using the Common Terminology Criteria for Adverse Events ver. 4.

Nausea was graded as follows: grade 1, loss of appetite without changes in eating habits; grade 2, decreased oral intake without significant weight loss, dehydration, or malnutrition; and grade 3, inadequate oral caloric or fluid intake, with tube feeding, total parenteral nutrition, or hospitalisation indicated.

Diarrhoea was graded as follows: grade 1, increase in the number of daily stool episodes by <4 relative to baseline or a mild increase in the daily ostomy output relative to baseline; grade 2, increase in the number of daily stool episodes by 4-6 relative to baseline or a moderate increase in the daily ostomy output relative to baseline; grade 3, increase in the number of daily stool episodes by ≥7 relative to baseline, incontinence, hospitalisation indicated, a severe increase in the daily ostomy output relative to baseline, or limited ability to perform self-care activities of daily living; and grade 4, life-threatening consequences or urgent intervention indicated.

| **Characteristics** |  |  | **Nintedanib only**  **n = 52** | **Nintedanib + Prednisolone**  **n = 25** | ***P*** |
| --- | --- | --- | --- | --- | --- |
| **Age** | |  | 71.56 ± 8.73 | 70.80 ± 8.71 | *0.722* |
| **Gender** | | Female / Male | 8 / 44 | 4 / 21 | *0.944* |
| **Smoking history** | | No / Yes | 7 / 45 | 4 / 21 | *0.766* |
| **PS** | | 0-1 / 2-4 | 37 / 15 | 18 / 7 | *0.939* |
| **GAP index** | | 0-5 / 6-9 | 31 / 21 | 15 / 10 | *0.975* |
| **BMI** | |  | 22.54 ± 3.95 | 23.28 ± 3.99 | *0.441* |
| **Initiation dose** | | 200 mg / 300 mg | 13 / 39 | 10 / 15 | *0.178* |
| **HRCT pattern** | | Definite / Possible | 36 / 16 | 17 / 8 | *0.913* |
| **PPI**  **(concomitant used)** | | No / Yes | 31 / 21 | 7 / 18 | 0.007 |
| **Intestinal drug (concomitant used)** | | No / Yes | 35 / 17 | 18 / 7 | 0.910 |

**Table S1-The difference in patient characteristics between patients who received nintedanib only and patients who received both nintedanib and prednisolone treatment**

PS: performance status, BMI: body mass index, HRCT: high resolution computed tomography, PPI: proton pump inhibitor

**Table S2- Association between gastrointestinal side effects and baseline pulmonary function in patients with idiopathic pulmonary fibrosis receiving nintedanib treatment**

| **Pulmonary function** | **Nausea** | |  | |
| --- | --- | --- | --- | --- |
|  | **No** | **Yes** | **t-value** | ***p*** |
|  | **n = 49** | **n = 16** |  |  |
| **FVC (L)** | 2.235 ± 0.115 | 1.992 ± 0.624 | 1.257 | *0.213* |
| **FVC (%)** | 68.78 ± 19.17 | 60.34 ± 18.54 | 1.677 | *0.098* |
|  | **n = 47** | **n = 11** |  |  |
| **DLco (L)** | 8.091 ± 3.083 | 6.038 ± 2.492 | 2.386 | *0.021* |
| **DLco (%)** | 34.67 ± 11.74 | 26.06 ± 10.91 | 2.546 | *0.014* |
|  | **Diarrhea** | |  | |
| **Pulmonary function** | **No** | **Yes** | **t-value** | ***p*** |
|  | **n = 42** | **n = 23** |  |  |
| **FVC (L)** | 2.285 ± 0.663 | 1.919 ± 0.781 | 2.016 | *0.048* |
| **FVC (%)** | 68.74 ± 17.63 | 61.15 ± 21.42 | 1.537 | *0.129* |
|  | **n = 42** | **n = 17** |  |  |
| **DLco (L)** | 7.785 ± 2.922 | 6914 ± 3.368 | 0.991 | *0.326* |
| **DLco (%)** | 33.07 ± 11.43 | 30.41 ± 13.60 | 0.763 | *0.449* |

FVC: focal vital capacity, DLco: diffusing capacity of the lungs for carbon monoxide
